# The first report of human illness associated with the Panola Mountain *Ehrlichia *species: a case report

**DOI:** 10.1186/1752-1947-2-139

**Published:** 2008-04-30

**Authors:** Will K Reeves, Amanda D Loftis, William L Nicholson, Alan G Czarkowski

**Affiliations:** 1 USDA-ARS-ABADRL, Department 3354, 1000 E University Ave, Laramie, WY 82071-2000, USA; 2 226 North Lincoln St, Laramie, WY 82070, USA; 3 Centers for Disease Control and Prevention, 1600 Clifton Rd NE, MS G-13, Atlanta, GA 30333, USA

## Abstract

**Introduction:**

Two species of *Ehrlichia *are known to cause human illness. Several other species have been discovered in ticks and animals, and recent reports suggest that some of these *Ehrlichia *species might be human pathogens. We report here the first association of a recently discovered pathogen, the Panola Mountain *Ehrlichia *species, with a case of human illness.

**Case presentation:**

A 31-year-old man from Atlanta, Georgia (GA) in the United States of America (USA) presented with a persistent sore neck of 3 weeks duration following a tick bite. DNA from the Panola Mountain *Ehrlichia *species, which was recently discovered in a goat in Georgia, was detected in an acute blood sample. Serologic testing was inconclusive. Polymerase chain reaction tests for other tick-borne diseases found in this region were negative. The patient rapidly improved in response to doxycycline therapy.

**Conclusion:**

Detection of *Ehrlichia *DNA in an acute blood sample meets the Centers for Disease Control and Prevention laboratory confirmation criteria for ehrlichiosis, and response to doxycycline provides supporting clinical evidence. The Panola Mountain *Ehrlichia *species, an emerging pathogen transmitted by ticks in the eastern USA, should be considered as a possible cause of tick-borne illness in this region.

## Introduction

*Ehrlichia *species are tick-transmitted intracellular bacteria closely related to *Anaplasma*, *Neorickettsia *and *Rickettsia*. *Ehrlichia chaffeensis *was first described as a human pathogen in 1986 and *E. ewingii *in 1996 [1], and recent evidence suggests that other species of *Ehrlichia *might also cause illness [2,3]. A new species of *Ehrlichia*, the Panola Mountain *Ehrlichia *species, was recently discovered in the USA [4]. Clinical signs of ehrlichial infection are often non-specific, and the most common signs are fever, headache, myalgia and malaise [1]. Laboratory diagnosis of ehrlichiosis depends on the detection of ehrlichiae in samples collected during the acute illness or on demonstration of a significant rise (four-fold or greater) in antibody titer between the acute and convalescent phases of the illness [1,5]. Serology is limited because acute serology alone is insufficient to diagnose infection, paired serology does not provide a diagnosis until 3 to 4 weeks after empirical treatment has been initiated, and only *E. chaffeensis *is available for serologic testing of humans [1]. Diagnosis of ehrlichiosis during the acute infection increasingly relies on polymerase chain reaction (PCR) [1,6]. According to the official case definition used by the Centers for Disease Control (CDC) and the USA National Notifiable Diseases System, a positive PCR reaction, with confirmation of the amplicon identity, is sufficient for laboratory confirmation of a case of human ehrlichiosis [5].

## Case presentation

On 23 September 2005, a 31-year-old Caucasian man from Atlanta, Georgia, USA presented with a complaint of neck soreness for 3 weeks. The patient reported hiking at Panola Mountain State Park in Georgia on 31 August 2005 and later removing a partially engorged nymphal tick from his upper arm on 3 September. The patient stored the tick in an empty vial at room temperature, and the tick was later identified as *Amblyomma americanum*. On 8 September, the patient began suffering from a persistent sore neck, characterized by musculoskeletal pain upon turning his head and insomnia due to pain. The pain was refractory to anti-inflammatory medications, including acetaminophen, aspirin and ibuprofen. Physical examination on 23 September was unremarkable. Pyrexia was not observed and no erythema or edema was noted at the site of the tick bite; however, the patient had taken 500 mg aspirin prior to examination. The patient was treated for a presumptive tick-borne illness with 100 mg of oral doxycycline twice daily for 10 days. The patient reported that neck soreness was improved by 48 to 60 hours after doxycycline therapy was initiated.

## Laboratory testing

Blood was drawn from the patient on 23 September for PCR testing for tick-borne diseases, on 26 September for a complete blood count (CBC) and acute serology, and on 15 October for convalescent serology. Whole blood from 23 September and sera from 26 September and 15 October were submitted to the CDC for tick-borne disease testing. The CBC was performed by Quest Diagnostics (Nichols Institute, Chantilly, VA), and CBC results (Table 1) were within the normal reference range for this laboratory.

**Table 1:** Complete blood count results for blood drawn on 26 September 2005

Hematocrit	46.7%
Red blood cell count	5.32 × 10^7^/μl
Hemoglobin	15.9 g/dl
Mean corpuscular volume	87.8 fl
Mean corpuscular hemoglobin	29.9 pg
Mean corpuscular hemoglobin concentration	34.1 g/dl
Red blood cell distribution width	12.7%
Platelet count	272,000/μl
White blood cell count	7500/μl
Absolute neutrophils (%)	4868 cells/μl (64.9%)
Absolute lymphocytes (%)	2243 cells/μl (29.9%)
Absolute monocytes (%)	233 cells/μl (3.1%)
Absolute eosinophils (%)	143 cells/μl (1.9%)
Absolute basophils (%)	15 cells/μl (0.2%)

For PCR testing, DNA was extracted from 100 μl of clotted blood and from the dead tick, using an IsoQuick Nucleic Acid Extraction Kit (ORCA Research Inc., Bothell, WA). We detected DNA from *Ehrlichia *using a genus-specific, hemi-nested PCR with the outer primers EC12A and HE3 [4], followed by a hemi-nested reaction using the 'Forward' primer [7] and HE3. DNA from *Rickettsia *species was detected using primer-1 and primer-2 [8]. We assessed the quality of the tick DNA using primers T1B and T2A [9]. Positive and negative controls were used for all assays and consisted of genomic DNA from *Rickettsia rickettsii, Ehrlichia ewingii *or distilled water. All PCR products were purified with a QIAquick PCR Purification Kit (Qiagen, Valencia, CA) and sequenced in duplicate using PCR primers and a BigDye Terminator v3.1 Cycle Sequencing Kit (Applied Biosystems, Foster City, CA). Sequences were determined using an ABI 3100 (Applied Biosystems). Primer sequences were removed and sequences assembled with Seqmerge (Accelrys, San Diego, CA).

Using the hemi-nested PCR, an amplicon from the 16S rRNA gene of an *Ehrlichia *species was obtained from the acute blood sample. The amplicon was sequenced, and the 361-bp sequence (GenBank accession number DQ217573) was 100% identical to the sequence reported from the Panola Mountain *Ehrlichia *species (*Ehrlichia *species P-Mtn, GenBank accession number DQ324367). The amplicon was not identical to sequences from any other species represented in GenBank. No DNA from *Rickettsia *was detected in the patient's blood. The tick was poorly preserved by the patient, and DNA could not be amplified from it.

For acute and convalescent serology, sera from 26 September and 15 October were tested using indirect immunofluorescence assays (IFA), performed as previously described [10], to detect antibodies against *Anaplasma phagocytophilum*, *Borrelia burgdorferi*, *Coxiella burnetii*, *Francisella tularensis*, *Rickettsia africae*, *R. akari*, '*R. amblyommii*', *R. conorii*, *R. parkeri*, *R. prowazekii*, *R. rickettsii *and *R. typhi*, and antibodies cross-reactive with *E. chaffeensis*. We could not test the patient's serum against the Panola Mountain *Ehrlichia *species because this emerging agent has not yet been cultured. Antibody was detected using isotype-specific goat antihuman immunoglobulin G (IgG) and human immunoglobulin M (IgM), labeled with fluorescein isothiocyanate (FITC) (KPL, Gaithersburg, Maryland). Prior to testing for IgM, sera were depleted of IgG by use of a recombinant Protein G kit (Rapi-Sep-M, Pan-Bio, Columbia, MD). Acute and convalescent samples were tested side-by-side, and positive, negative and diluent controls were assayed with the test samples and gave expected results. Serology did not support recent infection with any of the agents tested. With *E. chaffeensis *antigen, the patient's serum reacted with a small proportion of the organisms on the slide, as compared with positive control sera, and was considered cross-reactive in both acute and convalescent samples. Titers are expressed as the reciprocal of the last dilution exhibiting specific fluorescence and were as follows: IgM 32 (26 September), and IgG 16 (26 September) and IgG 32 (15 October). Convalescent IgM data were not available.

## Conclusion

We report that an emerging pathogen, the Panola Mountain *Ehrlichia *species, was detected in blood from a human patient following the bite of a nymphal *Amblyomma *that was probably acquired at Panola Mountain State Park in Georgia in the United States of America. The Panola Mountain *Ehrlichia *species was originally described from a goat fed upon by *A. americanum *collected at this park [4], but this is the first report associating the agent with human illness.

The Panola Mountain *Ehrlichia *species is genetically closely related to *E. ruminantium *and more distantly related to *E. chaffeensis *[4]. The patient exhibited myalgia for 3 weeks prior to presentation, had ehrlichemia, which was confirmed by DNA sequencing at presentation, and rapidly recovered after treatment with doxycycline. Although PCR and serological testing for other tick-borne agents was negative, suggesting that ehrlichiosis was the cause of illness, we cannot conclusively rule out the possibility that the patient's symptoms were caused by an unknown factor. Serological confirmation of infection with the Panola Mountain *Ehrlichia *species could not be obtained, with only a two-fold rise in IgG titer between the two serum samples. This might be due to the initiation of antibiotic therapy prior to optimal immune response or due to the lack of an appropriate antigen; antibodies against ehrlichial agents are often, but not always, cross-reactive with other species of *Ehrlichia *[3]. In this case, PCR testing of whole blood was of significantly greater diagnostic value than serological testing.

## Abbreviations

CBC: complete blood count; CDC: Centers for Disease Control; FITC: fluorescein isothiocyanate; IFA: immunofluorescence assays; IgG: immunoglobulin G; IgM: immunoglobulin M; PCR: polymerase chain reaction.

## Competing interests

The authors declare that they have no competing interests.

## Authors' contributions

WKR identified the tick, isolated DNA from the blood sample, performed sequencing reactions, analyzed data, and was a major contributor in writing the manuscript. ADL tested the DNA from the blood sample, recorded patient information, and was a major contributor in writing the manuscript. WLN performed serological tests and reviewed drafts of this manuscript. AGC was the attending physician for the patient and contributed to drafts, collecting clinical specimens, and patient treatment and observations. All authors read and approved the final manuscript.

## Consent

Written informed consent was obtained from the patient for publication of this case report. A copy of the written consent is available for review by the Editor-in-Chief of this journal.
